# Exploring the Nutritional Profile and Cost of Plant-Based Milk Alternatives Compared with Dairy Milk in the UK with Consideration of Environmental Impact Data

**DOI:** 10.1016/j.cdnut.2025.107436

**Published:** 2025-04-09

**Authors:** Gemma K Nowson, Rosalind Fallaize, Kate E Earl

**Affiliations:** Department of Clinical, Pharmaceutical and Biological Sciences, School of Health, Medicine and Life Sciences, University of Hertfordshire, Hatfield, United Kingdom

**Keywords:** milk, dairy, plant-based milk alternatives, milk substitutes, milk alternatives, food composition, food cost, ultraprocessed foods, nutritional profile, environmental impact

## Abstract

**Background:**

Plant-based milk alternatives (PBMA) are increasingly popular, driven by medical, environmental, or moralistic reasons or perceived health benefit.

**Objectives:**

This study aimed to compare the nutritional profile, cost, and environmental impact of all PBMA and dairy milk (DM) in the United Kingdom.

**Methods:**

Nutritional information, ingredients, and cost of PBMA (*n* = 191) and DM (*n* = 195) were systematically collected from the top 10 supermarkets. Published data on the environmental impact of foods were assessed. Milk was classified per 100 mL by energy (kcal), macronutrients (g), and micronutrients (mg, μg) and mean/median were compared between PBMA and DM. Further analysis stratified milks by DM fat profile. PBMA were categorized according to NOVA criteria. Cost per 1 L and environmental impact were compared for PBMA and DM.

**Results:**

PBMA with a similar fat profile to “semi-skimmed” milk had a significantly lower energy content (*P* < 0.001) and most (except coconut) had a significantly lower saturated fat content than DM. DM provided more protein, carbohydrate, total sugar, and salt and PBMA provided more fiber and total vitamin D. In total, 92% of nonorganic PBMA were fortified with ≥1 micronutrient; 87% with calcium, 34% iodine, 79% vitamin B_12_, and 56% vitamin B_2_. PBMA contained between 2% and 16.5% of the “main ingredient,” e.g. “oats.” Of nonorganic PBMA, 97% were classified as “ultraprocessed.” On mean, PBMA (£1.95/L) cost 64% more than DM (£1.19/L). Environmental analysis was conducted but not considered sufficiently robust to draw meaningful results (Appendix A).

**Conclusions:**

PBMA cannot be recommended as a nutritional replacement for DM, due to varying nutritional profiles. However, some PBMA will be more beneficial than others depending on an individual's health needs. Cow milk is cheaper than PBMA. Further understanding of the potential health impacts of consuming PBMA is warranted. There is a need for robust, primary research on the environmental impacts of foods.

## Introduction

Plant-based milk alternatives (PBMA) are made from plants such as grains, nuts, and seeds extracted in water and homogenized [1,2]. They are often fortified with nutrients and combined with other ingredients such as sugar, oil, flavorings, and stabilizers to make them more palatable and appealing to consumers [2,3], shelf-stable, and convenient [4].

In 2024, 34% of people used PBMA in a 3-mo period [5]. The per capita consumption of milk substitutes in the United Kingdom was 3.46 L in 2024, compared with 1.56 L in 2019, and is projected to be 4.93 L in 2029 [5]. In 2024, the volume of cow milk sold fell to 4644 L (from 4781 L in 2022) [6]. The purchase of dairy milk (DM) and milk products has decreased from 2978 mL per person per week in 1974 (∼2 glasses/d) to 1635 mL in 2022 (∼1 glass/d) [7].

There has been a cultural shift in the consumption of PBMA in recent years with individuals choosing PMBA for a variety of reasons including allergies/intolerances to DM, moralistic concerns, environmental motives, perceived health benefits. In the UK, cow milk allergy affects between 1.8% and 7.5% of infants in the first year of their life and although most children will outgrow their allergy by 5 y, a small number of people will continue to be allergic in adulthood [8,9]. A systematic review and meta-analysis estimated lactose-intolerance prevalence at 8% in the United Kingdom [10]; however, self-reported prevalence has been reported at 15% [11].

Individuals following a vegan diet commonly consume PBMA as an alternative to DM. The prevalence of people who follow a vegan diet excluding animal products including dairy in the United Kingdom is estimated at ∼4%–6% [12]. Reasons may include ethical concerns, environmental impact, religious or cultural beliefs or personal preference. Research has shown that a greater number of people are choosing PBMA to reduce their consumption of animal products [11].

The environmental impact of foods is a growing concern to many, leading some individuals to choose PBMA over DM [13]. Reducing carbon emissions to help mitigate climate change has been a key focus of United Kingdom policy in recent years [[14], [15], [16], [17]]. The Department for Environment, Food and Rural Affairs encourages healthier and more sustainable dietary choices. In the United Kingdom, the British Dietetic Association One Blue Dot campaign, provides guidance on sustainable eating which includes reducing dairy [18]. Several studies have investigated the environmental impact of milk. Silva's systematic review found that plant-based milk has a lower environmental impact than animal-based milk, with significantly lower greenhouse gas (GHG) emissions (0.021–3.85 kg CO_2_eq compared with 0.089–72.70 kg CO_2_eq per liter) and reduced land use, eutrophication, and acidification potential. However, almond milk had notably higher water use (59–6100 L per liter) [19]. Carlsson et al. [20] found that plant-based drinks, particularly soy and oat, had lower GHG emissions and land use than dairy, though almond milk’s water use varied across studies. In the United Kingdom, several large-scale life cycle assessment (LCA) studies have been completed analyzing the environmental impact of food and drink [[21], [22], [23]]. For example, Clark et al. [22] estimated the environmental impact of 57,000 foods using published environmental databases. They grouped “dairy alternatives” and found they had a lower environmental impact than “milk butter and eggs” introducing some bias. However, when compared with the environmental impact of meat sources such as beef and lamb, this difference appeared negligible [22]. Other studies included limited PBMA (oat, soya, and rice milk) [23] or compared “dairy” (milk, butter, and eggs) with “dairy alternatives” [22].

Many people consume PBMA due to perceived nutritional benefits. For example, a recent study found that the most frequently reported words in relation to PBMA were “healthy” or “nutritious” (52% of participants) suggesting that these products are associated with health benefits [24]. Research has shown that nutritional profile is dependent on the plant source used [[1], [25]].

Several studies in Europe, United States, Australia, and Canada have looked compared the nutritional composition of PBMA to DM [23,[27], [28], [29], [30], [31]]. A 2021 United Kingdom study [30] found DM to contain more energy, saturated fat, carbohydrates, protein, vitamin B_2_, vitamin B_12_, and iodine, and less fiber and free sugars, than PBMA overall (which included coconut, oat, rice, quinoa, soya, pea, almond, cashew, tiger nut, walnut, almond, and hazelnut). A more recent United Kingdom study [32] reported that soya, coconut, and almond milk had lower carbohydrates, sugars, calcium, iodine, and potassium than DM. However, this study was limited by a small sample milks (57 nondairy and 7 DMs) and did not capture the full range of nondairy milks available.

Cost is an important consideration for PBMA. The estimated annual cost of drinking cows’ milk versus replacing it with PBMA in the United Kingdom population across the lifespan was £48.00-88.07 for DM and£86.38–176.07 for PBMA [30]. Another study found that median prices were similar between dairy and nondairy milks [32]. However, in this rapidly growing market, data can quickly become out of date.

There is growing interest in ultraprocessed foods. The NOVA criteria were developed by Monteiro et al and put forward by the FAO of the United Nations and categorize food and drink in terms of their processing including the use of additives [33]. Ultraprocessed foods have been linked to a higher risk of noncommunicable diseases such as cancer, obesity, and diabetes [34]. However, the Scientific Advisory Committee on Nutrition (SACN) has noted that it remains uncertain whether these health risks are directly attributable to the processing itself or because these foods are typically high in energy, saturated fat, and/or free sugars [71]. Notably, existing studies have not specifically examined PBMA in this context. SACN have commissioned a joint working group of SACN and the Committee on Toxicity of Chemicals in Food, Consumer Products and the Environment (COT), to consider both toxicological and nutritional aspects associated with the consumption of PBMA in the general population in the United Kingdom [35].

Healthcare professionals see a variety of patients with different medical conditions, body composition, and activity levels. It is important for healthcare professionals to be able to draw on a strong evidence base and make recommendations on the most suitable DMs or PBMA depending on their nutritional status while also considering their individual preferences or concerns. It is also important to determine whether a population-wide change to PBMA may have impacts on health in the longer term.

There is a rapidly growing market for PBMA [36] with a wider range of PBMA available that previous work has not included. In addition, the nutritional profile of PBMA is evolving with new product formulations, processing methods, and fortification [37]. This study aims to investigate the nutritional profile (including the composition and processing of milks), cost (per L), and environmental impact of PBMA (sweetened and unsweetened) and dairy of all milks available in the top 10 United Kingdom supermarkets and aims to fill the gap in the current evidence base.

## Methods

### Sampling

An online search was completed for all DMs and PBMA from the top 10 supermarkets with the largest market share (Tesco, Sainsbury’s, Asda, Aldi, Morrisons, Lidl, The Co-operative, Waitrose, Iceland, and Ocado) [12]. One supermarket, Lidl, did not list details of their products online so a search was completed at the nearest local store. Nutritional profile per 100 mL and cost per 1 L were collected for all products across the 10 supermarkets between October 2023 and January 2024.

### Design and data collection

Product information was collected using back-of-pack labeling, supermarket websites, or supermarket shelves and cross-checked with the manufacturer’s websites (where given). In the case of discrepancies, or no nutritional information available, the manufacturer was contacted to confirm the correct information or back-of-pack labeling was used (required for 65 products across 27 manufacturers). Each milk was categorized into “dairy” or “PBMA” and then further categorized using the front-of-pack labeling into the different types of milks present in the data: cows, Jersey cows, lactose-free cows, goat, camel, kefir cows, soya, oat, oat kefir, rice, coconut, almond, hazelnut, hemp, cashew, pea, walnut, and combination (where milk contains >1 ingredient, e.g. pea and hemp). Where multiple supermarkets stocked the same milk, it was only counted once in the nutritional data. For example, Arla Cravendale Filtered Skimmed Milk was available at both Tesco and Sainsbury’s. Equally, the same product in different sizes was only counted once in the nutritional data. For example, Asda Skimmed milk was available in 1136 mL/568 mL/2273 mL. Sweetened and unsweetened varieties of PBMA were included. Flavored milks (e.g. chocolate cow milk or chocolate soya milk) and yogurt-style drinks were excluded from this study.

For PBMA and DM, the following nutritional information was collected per 100 mL: energy (kcal), total fat (g), saturated fat (g), carbohydrate (g), total sugar (g), fiber (g), protein (g), salt (g), and sodium (mg). For PBMA, the following micronutrients were collected per 100 mL: linoleic acid (g), alpha-linoleic acid (g), vitamin A (μg), vitamin B_1_ (mg), vitamin B_2_ (mg), vitamin B_3_ (mg), vitamin B_5_ (mg), vitamin B_6_ (mg), vitamin B_7_ (μg), vitamin B_9_ (μg), vitamin B_12_ (μg), vitamin C (mg), total vitamin D (μg), vitamin K1 (μg), vitamin K2 (μg), vitamin E (mg), calcium (mg), chloride (mg), copper (μg), iodine (μg), iron (mg), manganese (mg), magnesium (mg), phosphorus (mg), potassium (mg), selenium (μg), sulfur (mg), and zinc (mg). McCance and Widdowson’s “the composition of foods” tables [39] were used to collect micronutrient nutritional information for DMs as labels/websites did not provide this information. For micronutrients, PBMA were further categorized by “organic” and “nonorganic.” This is due to organic products having restrictions regarding fortification [40].

Product composition was investigated. All ingredients in PBMA, including the percentage of “main ingredient,” e.g. “oats” or “soya bean,” were recorded. All milks were classified by the NOVA criteria to determine their level of processing: *1*) unprocessed/minimally processed, *2*) processed culinary ingredients, *3*) processed foods or *4*) ultraprocessed. According to the NOVA classification [33,[41], [42], [43]], an “ingredients” list corresponding to each category of processing was devised and utilized to allow classification.

Cost per 1L for each milk was collected from supermarket websites or supermarket shelves (where not available online). Where multiple product sizes were available, an mean cost was calculated. Offers or any loyalty card prices were excluded.

Data were retrieved from Clark et al.’s [22] dataset which comprised “estimated” environmental impacts of 57,000 food and drink products (determined using a Montecarlo analysis). Products had a “total environmental impact score” which ranged from 0 (no impact) to 100 (highest impact) which combined GHG emissions (kgCO_2_e), scarcity-weighted water use (L), land use (m^2^) and aquatic eutrophication potential (gPO4eq). The total environmental impact score was collected for all milks in this study. Products also had “Fifty” (or median), “Lower twenty fifth” (or quartile 1), and “Upper seventy fifth” (or quartile 3) values for individual environmental impacts [GHG emissions, scarcity-weighted water use, land use, aquatic eutrophication potential, biodiversity, acidification (pH), and water use (L)] per 100 g which were also collected for all milks in this study. It should be noted that “mean” values were also available for individual environmental impacts; however, upon contacting Clark et al. they declared a large right skew in their data, so recommended using median, Q1 and Q3 rather than mean values.

Where there were multiple data points for the same milk, a median was calculated. Blanks in the dataset (denoted as “NA”) were confirmed as missing data and left as blanks. Missing or “zero” data in the dataset were checked and confirmed and matched accordingly.

### Outcomes

The first outcome was to compare the nutritional profile of PBMA and DMs. The second outcome was to investigate product composition by: *1*) comparing the median (and IQR) percentage of the main ingredient in each type of PBMA and the percentage of milks within each subtype of PBMA that contained added ingredients and *2*) classifying PBMA as per the NOVA criteria. The third outcome was to compare the environmental impact for PBMA and DM and the fourth outcome was to compare cost per 1 L for PBMA and DMs.

### Statistical analysis

The mean energy (kcal/100 mL), total fat (g/100 mL), saturated fat (g/100 mL), carbohydrate (g/100k mL), total sugar (g/100 mL), fiber (g/100 mL), protein (g/100 mL), and salt (g/100 mL) were compared between all 18 sub milk types.

Dairy milks had a multimodal distribution for energy, total fat, and saturated fat (due to varying and discrete fat profiles). Data were recategorized by the following “fat profiles” to make comparisons between milk types more meaningful: 0.1–0.5 g/100 mL (similar to fat profile of skimmed), 0.6–1.4 g/100 mL (between fat profile of skimmed and semi-skimmed), 1.5–2 g/100 mL (similar to fat profile of semi-skimmed), 2.1–3.4 g/100 mL (between fat profile of semi-skimmed and whole), 3.5–4 g/100 mL (similar to fat profile of whole), 4.1–4.7 g/100 mL (between fat profile of whole and Jersey), and 4.8–5.7 g/100 mL (similar to fat profile of Jersey). Data were checked for normality and Mann–Whitney *U* tests (for non-normality within groups) were used to determine whether there were differences between PBMA and DM across each fat profile category. Categories with sample sizes <5 were excluded from statistical analysis (these were 0.1–0.5 g/100 mL, 0.6–1.4 g/100 mL, 4.1–4.7 g/100 mL, and 4.8–5.7 g/100 mL).

For carbohydrate, total sugar, fiber, protein, and salt, data were checked for normality and a Kruskal–Wallis test (for non-normality within the groups) was used to determine whether there were differences between PBMA and DMs and between milk subtypes. Milk subtype categories were excluded from statistical testing where sample sizes were <5 or if they were not available in all supermarkets (camel, rice, hazelnut, cashew, hemp, pea, walnut, Jersey, goats, kefir, oat kefir, coconut, and combination milks).

The proportion of PBMA that are fortified with micronutrients was calculated. Of those milks that are fortified, the mean content of these micronutrients was compared between PBMA milks and DMs [39]. Data were checked for normality and 1 sample Wilcoxon signed rank tests (for non-normality within groups) or one-sided *t*-tests (for normality within the groups) tested for statistically significant differences between mean/median values. Values given for sodium were converted to “salt.” Vitamin A was omitted due to the reference level across DMs being highly variable.

The percentage of PBMA that are categorized as unprocessed, minimally processed, processed, and ultraprocessed according to the NOVA criteria was calculated. The percentage of “main ingredient” in PBMA subtypes was checked for normality and the median percentage and IQR were calculated each (due to non-normality within the PBMA subtypes). The number of milks containing added ingredients within each PBMA subtype was calculated and presented as a percentage.

The median cost of DM was compared with PBMA. Data were checked for normality and a Mann–Whitney *U* test (for non-normal data) tested whether there were significant differences in cost between PBMA and dairy. Milk subtypes were excluded from the analysis where sample size was <5 (Jersey, cashew, combination, hazelnut, pea, rice, and walnut).

Using the cleaned dataset, a median (and IQR) total environmental impact score was calculated for PBMA and DM and for each subtype of milk. Data were checked for normality and a Mann–Whitney *U* test was used to determine whether there were differences between PBMA and DM and Kruskal–Wallis test was used to determine whether there were significant differences between milk subtypes. Milk subtypes were excluded from the analysis where sample size was <5 (Jersey, cashew, combination, hazelnut, pea, rice, and walnut).

Median “Fifty” and median IQR (“upper seventy fifth” (quartile 3)—“lower twenty fifth” (quartile 1)) “greenhouse gas emissions,” “scarcity-weighted water use,” “land use,” “aquatic eutrophication potential,” “acidification,” and “water use” per 100 g of product were calculated for PBMA and DM and for each subtype of milk.

*P* values were considered statistically significant if <0.05. Data were analyzed using SPSS version 23.

## Results

**FIGURE 1:**
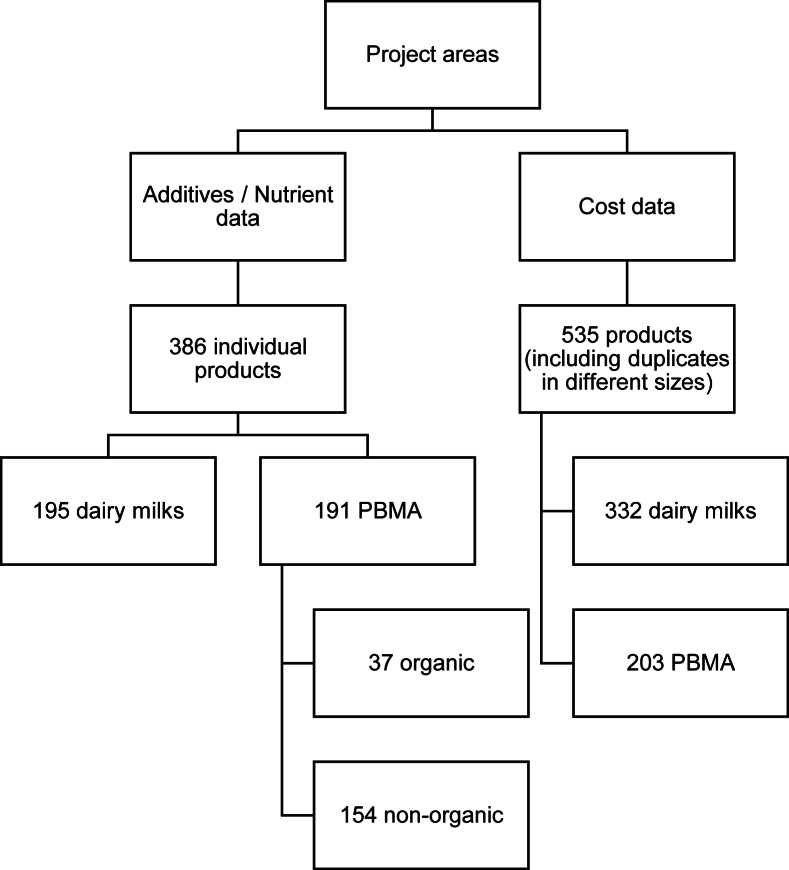
Summary of data collected.

Figure 1 shows a summary of the data collected.

### Nutritional profile

Table 1 shows the median energy (kcal/100 mL), total fat (g/100 mL), saturated fat (g/100 mL), carbohydrate (g/100k mL), total sugar (g/100 mL), fiber (g/100 mL), protein (g/100 mL) and salt (g/100 mL) plus the IQRs for each subtype of milk. It also presents the *P* value indicating statistically significant differences in median nutrient between milk subtypes.

**TABLE 1:** Median (and IQR) energy (kcal/100 mL), total fat (g/100 mL), saturated fat (g/100 mL), carbohydrate (g/100k mL), total sugar (g/100 mL), fiber (g/100 mL), protein (g/100 mL), and salt (g/100 mL) for each subtype of milk.

Milk	*N*	Energy (kcal/100 mL)	Total fat (g/100 mL)	Saturated fat (g/100 mL)	Carbohydrate (g/100 mL)	Sugars (g/100 mL)	Fiber (g/100 mL)	Protein (g/100 mL)	Salt (g/100 mL)
		Median (IQR)							
Dairy	195b	50 (27)	1.8 (3.1)	1.1 (2.2)	4.7 (0.2)	4.7 (0.2)	0 (0)	3.5 (0.2)	0.11 (0.01)
Camel	1b	53^1^	2.8^1^	1.7^1^	4.4^1^	4.2^1^	n.d	3^1^	0.19^1^
Cows	157^2^	50 (28)	1.8 (3.1)	1.1 (2.2)	4.8 (0.2)	4.8 (0.2)	0 (0)	3.5 (0.2)	0.11 (0.01)
Jersey	6^2^	79.5 (7)	4.95 (0.38)	3.4 (0.3)	4.6 (0.3)	4.6 (0.3)	0.5 (0.38)	3.65 (0.125)	0.1 (0.125)
Lactose free	21^2^	41 (15)	1.7 (1.63)	1 (0.9)	3.3 (0.7)	3.3 (0.7)	0.5 (0.5)	3.3 (0.2)	0.1 (0.025)
Goats	7^2^	45 (18)	1.7 (1.9)	1.2 (1.5)	4.3 (0.1)	4.3 (0.1)	0 (0)	3 (0.2)	0.1 (0)
Kefir	3^2^	59.00	2.9	2.10	4.60	3.50	n.d	3.20	0.1
PBMA	191	39 (24)	1.5 (0.9)	0.24 (0.2)	3.2 (5.4)	2.5 (2.8)	0.5 (0.3)	0.8 (1.16)	0.1 (0.03)
Almond	37^2^	18 (12)	1.4 (0.4)	0.1 (0.2)	1.3 (2.45)	0.35 (2.5)	0.5 (0.2)	0.6 (0.3)	0.135 (0.055)
Cashew	2	31.00	2.35	0.4 (0)	1.5 (0)	0.10	0.40	0.9 (0)	0.11
Coconut	25	29 (28)	1.4 (1.35)	1.2 (1.1)	2.1 (2.05)	1.9 (1.6)	0.4 (0.35)	0.5 (0.85)	0.1 (0.05)
Combination	10^2^	49.5 (12)	1.45 (0.85)	0.3 (1.1)	8 (3.075)	3.2 (2.9)	0.5 (0.35)	0.55 (0.675)	0.08 (0.05)
Hazelnut	4	29 (32)	1.65 (1)	0.2 (0)	3.15 (9.55)	3.15 (3.8)	0.3 (0.15)	0.45 (0.175)	0.115 (0.05)
Hemp	1	26^1^	2.7^1^	0.3^1^	0.9^1^	0.2^1^	0.1^1^	1^1^	0.05^1^
Oat	65^2^	48 (11)	1.8 (1.1)	0.2 (0.1)	6.9 (1.3)	3.2 (1)	0.65 (0.3)	0.8 (0.6)	0.1 (0.02)
Oat kefir	1	61^1^	3.4^1^	2.2^1^	7.4^1^	3.9^1^	0.3^1^	0^1^	0.1^1^
Pea	3	25.00	1.96	0.28	0.42	0.00	0.00	2.00	0.10
Rice	2	62.50	0.95	0.15	13.05	6.75	0.25	0.35	0.09
Soya	40^2^	33.5 (12)	1.8 (0.55)	0.3 (0.1)	2.5 (2)	2.2 (2.2)	0.5 (0.2)	3.25 (1.2)	0.105 (0.05)
Walnut	1	25^1^	1.3^1^	0.2^1^	2.9^1^	2.8^1^	0.1^1^	0^1^	0.1^1^
*P* value (comparing dairy and PBMA)	/	/	/	/	0.274	<0.0001	<0.0001	<0.0001	0.750
*P* value (comparing subtype milk)	/	/	/	/	<0.0001	<0.0001	<0.0001	<0.0001	<0.0001

*P* values indicate significant differences between subtypes of milk (milks with sample sizes <5 or if they were not available in all supermarkets were excluded from the analysis) and between dairy and PBMA.

Abbreviations: IQR, interquartile range; PBMA, plant-based milk alternative; n.d., no data.

1 Data represents actual value rather than median.

2 Some milks had incomplete nutritional information.

### Energy

Table 1 shows the milk with the highest energy is Jersey milk with a median value of 79.5 kcal/100 mL and the milk with the lowest energy is almond with a median value of 18 kcal/100 mL. All PBMA are lower in energy than DMs except for rice milk.

Supplemental Table 1 displays the median (and IQR) energy (kcal/100 mL) for dairy and PBMA according to different fat profiles and Supplemental Figure 1 shows scatterplots illustrating the spread of the data. Dairy milks generally have 3 “discrete” fat profiles, and PBMA vary more widely between the fat profiles. There were significant differences in the median energy between dairy and PBMA in the “semi-skimmed” fat profile (difference of 7 kcal/100 mL) (*P* < 0.00) and the “whole” fat profile (difference of 16.5 kcal/100 mL) (*P* < 0.001) with PBMA providing lower energy than dairy.

### Total fat

Table 1 shows the milk with the highest total fat content is Jersey milk with a median value of 4.95 g/100 mL followed by oat kefir, kefir, camel, and cashew milks. The milk with the lowest median total fat content is rice milk (0.95 g/100 mL). Supplemental Table 1 shows the varying total fat content (kcal/100 mL) within milk subtypes according to the fat profiles (with sample sizes above 5) and Supplemental Figure 2 shows scatterplots illustrating the spread of the data. There were no significant differences in the median total fat between dairy and PBMA across all fat profiles.

### Saturated fat

Table 1 shows the milk with the highest saturated fat content is Jersey milk with a median value of 3.4 g/100 mL, followed by oat kefir, kefir, and coconut milks. The milk with the lowest median saturated fat was almond milk (0.10 g/100 mL). Supplemental Table 1 shows the varying saturated fat content (kcal/100 mL) within milk subtypes according to the fat profiles (with sample sizes above 5) and Supplemental Figure 3 shows scatterplots illustrating the spread of the data which is again wider in PBMA. There were significant differences in the median saturated fat between dairy and PBMA in the “semi-skimmed” fat profile (difference of 0.9 g/100 mL) (*P* < 0.000) and the “between semi-skimmed and whole” category (difference of 1.7 g/100 mL) (*P* < 0.001), with PBMA being lower.

### Carbohydrate

The median carbohydrate content of DM was 4.7 g/100 mL compared with 3.2 g/100 mL for PBMA; however, this was not significant (*P* = 0.274). From Table 1, the milk with the highest carbohydrate content was rice milk with a median value of 13.05 g/100 mL, followed by combination (where a milk contains >1 ingredient, e.g. pea and hemp) and oat milk. The milks with the lowest median carbohydrate content are pea (0.42 g/100 mL) and hemp (0.9 g/100 mL). There were significant differences in the carbohydrate content of the subtypes of milk (*P* < 0.0001). Pairwise comparisons show that cows were significantly higher than almond (*P* < 0.0001), oat was significantly higher than almond (*P* < 0.0001), cows were significantly higher than soya (*P* < 0.0001), oat was significantly higher than soya (*P* < 0.0001), cows were significantly higher than lactose free (*P* < 0.0001), oat was significantly higher than lactose free, and oat was significantly higher than cows.

### Total sugar

The median total sugar content of DM was 4.7 g/100 mL compared with 2.5 g/100 mL for PBMA which was significant (*P* < 0.0001). Table 1 shows that the milk with the highest total sugar content was rice milk with a median value of 6.75 g/100 mL. The milks with the lowest median total sugar were pea (0 g/100 mL), cashew (0.1 g/100 mL), and hemp (0.2 g/100 mL). There were significant differences in the total sugar content of the subtypes of milk (*P* < 0.0001). Pairwise comparisons show that oat was significantly higher than soya (*P* = 0.003), lactose free was significantly higher than soya (*P* = 0.004), cows were significantly higher that soya (*P* < 0.0001), oat was significantly higher than almond (*P* = 0.003), lactose free was significantly higher than almond (*P* = 0.004), cows were significantly higher than oat (*P*,0.0001) and cows were significantly higher than lactose free (*P* < 0.0001).

### Fiber

The median fiber content of DM was 0 g/100 mL compared with 0.5 g/100 mL for PBMA which was significant (*P* < 0.0001). Table 1 shows that cow milk and pea milk did not contain any fiber, compared with the other milks, and oat milk had the highest median value (0.65 g/100 mL). There were significant differences in the fiber content of the subtypes of milk (*P* < 0.0001). Pairwise comparisons show that almond was significantly higher than cows (*P* < 0.0001), soya was significantly higher than cows (*P* < 0.0001), oat was significantly higher than cows (*P* < 0.0001), and oat was significantly higher than lactose free (*P* < 0.0001).

### Protein

The median protein content of DM was 3.5 g/100 mL compared with 0.8 g/100 mL for PBMA which was significant (*P* < 0.0001). The only PBMA with a similar protein content to DM is soya milk with cow milk having a median value of 3.5 g/100 mL compared with 3.25 g/100 mL for soya. All other PBMA were lower with median values between 0 and 2.0 g/100 mL (Table 1). There were significant differences in the protein content of the subtypes of milk (*P* < 0.0001). Pairwise comparisons show that soya was significantly higher than almond, lactose free was significantly higher than almond (*P* < 0.0001), cows were significantly higher than almond (*P* < 0.0001), soya was significantly higher than oat (*P* < 0.0001), lactose free was significantly higher than oat (*P* < 0.0001), cows were significantly higher than oat (*P* < 0.0001), and cows were significantly higher than soya (*P* < 0.0001).

### Salt

The median salt content of DM was 0.11 g/100 mL compared with 0.1 g/100 mL for PBMA but this difference was not significant (*P* < 0.750). According to Table 1, camel milk had the highest salt content (0.19 g/100 mL) per 100 mL. Of PBMA, the milk with the highest salt content was almond milk (0.14 g/100 mL). There were significant differences in the salt content of the subtypes of milk (*P* < 0.0001). Pairwise comparisons showed that lactose free was significantly higher than cows (*P* = 0.001), almond was significantly higher than lactose free (*P* < 0.0001), almond was significantly higher than soya (*P* = 0.001), almond was significantly higher than oat (*P* < 0.0001), and almond was significantly higher than cows (*P* 0.0001).

### Micronutrients

Table 2 shows the number and percentage of “nonorganic” PBMA that are fortified with micronutrients according to the back-of-pack labeling. It also shows for those that are fortified the median (and IQR) amount (and IQR) of micronutrient, and the dairy reference value (calculated from the mean of skimmed, semi-skimmed and whole milk from McCance and Widdowson food composition tables) for comparison [39]. *P* values show significant differences between PBMA and dairy for each micronutrient. Table 2 also shows the dietary reference values for soya milk (sweetened and unsweetened) compared with the back-of-pack labeling for the median soya milks collected.

**TABLE 2:** Median micronutrient content in nonorganic PBMA and dairy milk.

	Vitamin A (μg/100 mL)	Vitamin B_2_ (mg/100 mL)	Vitamin B_6_ (mg/100 mL)	Vitamin B_9_ (μg/100 mL)	Vitamin B_12_ (μg/100 mL)	Vitamin C (mg/100 mL)	Total vitamin D (μg/100 mL)	Vitamin E (mg/100 mL)	Calcium (mg/100 mL)	Chloride (mg/100 mL)	Iodine (μg/100 mL)	Iron (mg/100 mL)	Manganese (mg/100 mL)	Magnesium (mg/100 mL)	Phosphorus (mg/100 mL)	Potassium (mg/100 mL)	Zinc (mg/100 mL)
*N* (PBMA = 154)	9	86	1	7	122	4	128	22	134	1	52	6	1	2	2	5	3
% nonorganic PBMA containing micronutrient	6	56	1	5	79	3	83	14	87	1	34	4	1	1	1	3	2
Median nonorganic PBMA (IQR)	36 (79.74)	0.21 (0)	2.5^1^	26.4 (14.3)^2^	0.38 (0)	10.5 (3)	0.75 (0.05)	1.8 (0)	120 (0)	84^1^	22.5 (7.5)	0.87 (0.72)^2^	0.2a	13 (0)	25 (0)	184.1 (114.16) 2	0.60
Dairy reference value	19.67	0.23	0.06	8.67	0.87	1.67	Trace	0.05	124.33	87.33	27.33	0.03	/	11.33	96.00	161.33	0.47
*P* value	4	<0.001	3	0.017	0.000	0.063	/	<0.001	0.000	3	0.041	0.036	/	3	3	0.679	0.102

Dairy milk also contains small amounts of vitamins B_1_, B_3_, B_5_, B_7_, K1, and selenium but these were not present in PBMA. Overall, 92% of nonorganic PBMA were fortified with ≥1 micronutrient. Including organic milks, only 78% of PBMA were fortified with ≥1 micronutrient. Micronutrient fortification varied between nonorganic PBMA milk subtypes. 97% soya, 95% coconut, 25% combination, 100% hazelnut, 0% hemp, 96% oat, 100% oat kefir, 100% pea, 97% soya, and 100% walnut.

Abbreviations: IQR, interquartile range; PBMA, plant-based milk alternative.

1 Data represent actual value.

2 Data presented are mean/ standard deviation.

3 Unable to carry out significance testing as sample size too low.

4 Unable to carry out significance testing due to highly variable content of vitamin A in dairy milks.

From Table 2, compared with the reference values for dairy, for those nonorganic PBMA that are fortified, there were significant differences in, mean vitamin B_9_, median vitamin E, and mean iron (PBMA values are higher than in dairy) and median vitamin B_2_, median vitamin B_12_, median calcium, and median iodine (dairy values were higher than PBMA).

### Product composition

**FIGURE 2:**
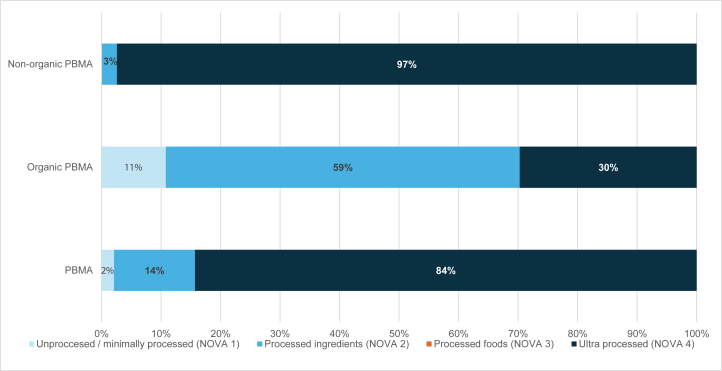
Percentage of milks that fall into NOVA categories. NOVA 1 included starchy roots, fruit extract, NOVA 2 included oil, sugar, salt, sea salt, tapioca starch, potassium iodide, NOVA 3 included starches, NOVA 4 included emulsifier, color, flavoring, fructose, maltodextrin, fruit juice concentrate, protein, fiber, stabilizers, and acidity regulators. PBMA, plant-based milk alternative.

Table 3 shows the NOVA classifications and the corresponding added ingredients. For example, if a product contains emulsifier or color, it is classified as NOVA 4: ultraprocessed. The literature [[35], [43], [44], [45]] did not state which category the following ingredients were in: tapioca starch, acidity regulators, stabilizers, and upon contacting the authors for further guidance, they were unable to clarify so the team made an informed decision. Stabilizers (act as a thickener) and acidity regulators (change the taste of foods) were classified by the team as NOVA 4 and potassium iodide (similar to salt) and tapioca syrup (similar to maple syrup) were classified by the team as NOVA 2. Figure 2 shows for all PBMA, organic and nonorganic PBMA, the percentage of milks fall into each NOVA category. A total of 97% of nonorganic PBMA were ultraprocessed. According to NOVA criteria, DM is classified as NOVA 1 (unprocessed).

**TABLE 3:** NOVA score, NOVA category, and corresponding added ingredients which determine the level of processing.

NOVA score	NOVA category	Added ingredients
1	Unprocessed/minimally processed	Starchy roots, fruit extract
2	Processed culinary ingredients	Oil, sugar, salt, sea salt, tapioca starch, potassium iodide
3	Processed foods	Starches
4	Ultraprocessed	Emulsifier, color, flavoring, fructose, maltodextrin, fruit juice concentrate, protein, fiber, stabilizers, acidity regulators

Table 4 shows the median (and IQR) percentage of “main ingredient” in PBMA subtypes (e.g. “oats” or “soya bean”) and the percentage of milks within each PBMA subtype that list added ingredients on the label. From Table 4, rice milk had the highest median percentage of “rice” and almond and walnut milks had the lowest median percentage of those nuts, respectively. DM did not list ingredients on the back-of-pack labeling. In terms of added ingredients to PBMA the most commonly added ingredient is salt (73%–100% of PBMA). Other ingredients such as added sugar/sweeteners, acidity regulators, flavoring, stabilizers, and emulsifiers were commonly added to PBMA. It was noted (although not recorded) that some PBMA included words like “semi” or “light” on the front-of-pack labeling, which could lead to a presumed energy content.

**TABLE 4:** Median and IQR percentage of main ingredient in each type of PBMA and percentage of milks within each subtype of PBMA that contain added ingredients.

	Type of PBMA												
	Almond	Cashew	Coconut	Combination	Hazelnut	Hemp	Oat	Oat kefir	Pea	Rice	Soya	Walnut	All PBMA
Median (IQR) % main ingredient													
*N*	37	2	25	10	4	1	65	1	3	2	40	1	191
Main ingredient	2 (0.3)	5 (0)	9.6 (4.25)	10.25 (5.6)	2.8 (1.88)	4^1^	10 (0.1)	11^1^	2.4	17	8.35 (2)	2^1^	N/A
% of milks with ingredient added													
Total sugar	35	0	16	0	50	0	11	0	33	0	53	100	26%
Sugar	22	0	16	0	50	0	6	0	0	0	30	0	16%
Raw cane sugar	5	0	0	0	0	0	0	0	0	0	5	100	3%
Agave syrup	3	0	0	0	0	0	0	0	33	0	0	0	1%
Fructose	5	0	8	0	0	0	0	0	0	0	5	0	3%
Maltodextrin	0	0	0	0	0	0	0	0	0	0	0	0	6%
Tapioca syrup	0	0	0	0	0	0	0	0	0	0	3	0	1%
Total salt	92	100	100	100	100	100	98	100	100	100	73	100	92%
Salt	19	0	12	10	0	0	52	100	67	50	25	100	31%
Sea salt	73	100	88	90	100	100	46	0	33	50	48	0	61%
Total oil	8	0	20	30	25	0	91	100	100	100	8	0	42%
Oil (rapeseed)	0	0	16	20	0	0	46	0	100	0	0	0	20%
Sunflower oil	8	0	4	10	25	0	51	100	33	100	8	0	24%
Shea	0	0	0	0	0	0	0	0	0	0	5	0	1%
Sunflower seed extract	0	0	0	20	0	0	0	0	0	0	0	0	1%
Total acidity regulator	76	0	80	20	50	100	83	100	100	0	88	100	77%
Acidity regulator: dipotassium phosphate	0	0	16	0	0	100	34	0	67	0	0	0	15%
Acidity regulator: potassium phosphate	11	0	8	0	0	0	15	0	0	0	23	0	13%
Acidity regulator: potassium citrate	5	0	0	0	0	0	0	0	0	0	0	0	1%
Acidity regulator: potassium carbonate	0	0	0	10	0	0	5	0	33	0	0	0	3%
Acidity regulator and mineral: calcium citrate	3	0	0	0	0	0	0	0	0	0	0	0	1%
Acidity regulator and mineral: calcium carbonate	22	0	12	10	0	0	51	0	100	0	28	0	31%
Acidity regulator and mineral: tricalcium phosphate	22	0	52	0	50	0	20	0	0	0	28	0	25%
Acidity regulator and mineral: calcium phosphate	24	0	24	0	0	0	14	100	67	0	30	100	21%
Mineral: potassium Iodide	19	0	12	0	0	0	28	0	0	0	30	0	21%
Total flavoring	24	0	68	20	0	0	14	100	67	0	55	100	33%
Flavoring	22	0	68	20	0	0	14	100	67	0	55	100	32%
Natural aroma	3	0	0	0	0	0	0	0	0	0	0	0	1%
Total stabilizer	81	0	84	20	50	100	48	100	33	0	93	100	66%
Gellan gum (E418)	78	0	84	20	50	100	45	0	33	0	93	100	64%
Guar gum	14	0	40	20	0	0	2	0	33	0	0	0	10%
Locust bean gum	24	0	0	0	50	0	0	0	0	0	3	0	6%
Tara gum	3	0	0	0	0	0	0	0	0	0	0	0	1%
Xanthan gum	19	0	36	10	0	0	2	0	0	0	0	0	9%
Carregeenan	3	0	12	0	0	0	0	0	0	0	0	0	2%
Carob gum	3	0	0	0	0	0	0	0	0	0	0	0	1%
Cellulose gum	0	0	16	0	0	0	0	0	0	0	3	0	3%
Carboxy methyl cellulose	19	0	0	0	0	0	0	0	0	0	3	0	4%
Acacia gum	0	0	0	0	0	0	2	0	0	0	0	0	1%
Pectin	0	0	0	0	0	0	0	100	0	0	0	0	1%
Total emulsifier	70	0	48	0	50	100	2	0	0	0	5	100	24%
Sunflower lecithin	46	0	0	0	0	100	0	0	0	0	3	0	10%
Lecithins	19	0	0	0	50	0	2	0	0	0	0	0	5%
Rapeseed lecithin	5	0	0	0	0	0	0	0	0	0	0	0	1%
Sucrose esters of fatty acids	0	0	48	0	0	0	0	0	0	0	3	100	7%
Color (carotene)	0	0	20	0	0	0	0	0	0	0	0	0	3%
Total flour	3	0	12	0	0	0	0	100	0	0	0	0	3%
Organic carob seed flour	3	0	0	0	0	0	0	0	0	0	0	0	1%
Rice flour	0	0	12	0	0	0	0	100	0	0	0	0	2%
Total juice	0	0	24	0	0	0	0	100	0	0	3	0	4%
Concentrated apple juice	0	0	20	0	0	0	0	0	0	0	3	0	3%
Concentrated grape juice	0	0	4	0	0	0	0	0	0	0	0	0	1%
Lemon concentrate	0	0	0	0	0	0	0	100	0	0	0	0	1%
Total fruit extract	0	0	0	0	0	0	0	100	0	0	10	0	3%
Apple	0	0	0	0	0	0	0	100	0	0	10	0	3%
Carob	0	0	0	0	0	0	0	100	0	0	0	0	1%
Grape	0	0	0	0	0	0	0	100	0	0	0	0	1%
Total fiber/starch	5	0	0	0	0	0	28	100	0	0	20	0	15%
Soluble maize fiber	0	0	0	0	0	0	2	0	0	0	0	0	1%
Chicory root fiber	0	0	0	0	0	0	11	0	0	0	2	0	7%
Soluble corn fiber	0	0	0	0	0	0	6	0	0	0	3	0	3%
Oat fiber	3	0	0	0	0	0	0	0	0	0	0	0	1%
Tapioca starch	3	0	0	0	0	0	0	100	0	0	0	0	1%
Inulin	0	0	0	0	0	0	3	0	0	0	13	0	4%
Total protein	3	0	12	20	0	0	17	0	0	0	0	0	9%
Pea protein	0	0	0	0	0	0	14	0	0	0	0	0	5%
Broad bean protein	0	0	0	0	0	0	3	0	0	0	0	0	1%
Hemp protein	0	0	0	10	0	0	0	0	0	0	0	0	1%
Sunflower seed protein	3	0	0	0	0	0	0	0	0	0	0	0	1%
Faba bean protein	0	0	12	10	0	0	0	0	0	0	0	0	2%
Nutritional yeast	0	0	0	20	0	0	0	0	0	0	0	0	1%
Hatomugi	0	0	0	0	0	0	0	0	0	0	3	0	1%

Abbreviations: IQR, interquartile range; N/A; PBMA, plant-based milk alternative.

1 Represents actual data where a median could not be calculated.

### Cost of milks

**FIGURE 3:**
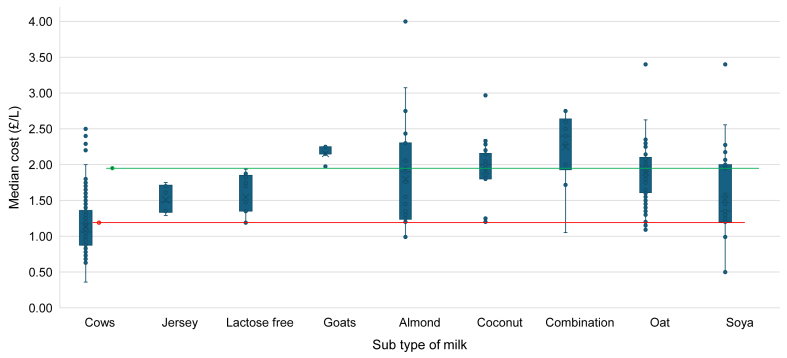
Box and whisker plot showing the mean cost (£/L) of each subtype of milk. Combination milk refers to where a milk contains >1 ingredient, e.g. pea and hemp. The cost (£) of dairy milk is shown in red [median £1.19/1L (IQR: 0.62)], and cost (£) of PBMA is shown in green [median £1.95 per 1Ll (IQR: 0.82)]. Those with sample sizes <5 were omitted from the figure (kefir, rice, hazelnut, cashew, pea, camel, hemp, oat kefir, and walnut). IQR, Interquartile range; PBMA, plant-based milk alternative.

Figure 3 shows a boxplot of the median cost (£/L) of each subtype of milk with sample sizes >5. It also shows for reference, the median cost (£) of DM (shown by the red line), and the median cost (£) of PBMA (shown by the green line). Median costs were calculated across a range of product sizes.

Camel milk was the most expensive milk at £13.83/L (only available in a 250 mL serving), followed by oat kefir (£7.40/L) and kefir (£5.20/L, IQR: 4.7–6.6). Cow milk was the cheapest at a median cost of £1.06/L (IQR: 0.88–1.36) (Supplemental Table 2). The median cost of DM is £1.19/1 L (IQR: 0.91–1.50), compared with PBMA which is £1.95 per 1 L (IQR: 1.35–2.15), and this difference is statistically significant (*P* < 0.001).

### Environmental impact

An analysis of the environmental impact was completed on 190 milks of those collected in the main study (123 dairy and 67 PBMA including duplicates in different sizes) using the Clark et al. data set [22]. This equated to around 36% of all milks (37% DMs and 33% PBMA). Given the limitations of the data (duplicate data and/or missing values) combined with the limitations of LCA methods (explained in the Discussion section), the analysis was considered not sufficiently robust to draw meaningful conclusions. For full transparency, the analysis has been presented in an Appendix entitled: An exploration into the environmental impact of PBMAs compared with DM in the United Kingdom.

## Discussion

This study aimed to investigate the nutritional profile, cost and environmental impact of PBMA and DM in the United Kingdom. The findings indicated substantial variability in the nutritional profiles of different PBMAs, which overall, were not equivalent to that of DM. Categorisation of PBMA by fat profile identified PBMA with a similar fat profile to “semi-skimmed” milk had a significantly lower energy content than DM and the majority of PBMA had a significantly lower saturated fat content than DM. DM provided more protein, carbohydrate, and total sugar than PBMA; however, PBMA provided more fiber and total vitamin D than DM. These data suggest potential benefits of PBMA consumption in specific populations. The nutritional profile of PBMA is largely influenced by the composition of the milks. Analysis revealed high levels of fortification in PBMA with 92% of nonorganic PBMA fortified with ≥1 micronutrient thus impacting the micronutrient profile. Overall, PBMA had higher mean vitamin B_9_, median vitamin E, and mean iron whereas DM had higher median vitamin B_2_, median vitamin B_12_, median calcium, and median iodine. PBMA composition is varied with the “main ingredient,” for example “oats” accounting for between 2-16.5% of the product. PBMA undergo higher levels of processing to maintain milk taste, texture, shelf life and nutritional value. This study revealed 97% of nonorganic PBMA were classified as “ultraprocessed.” under the NOVA classification. Cost analysis revealed PBMA are generally more expensive than DM.

### Nutritional profile

This study found that all PBMA are lower in energy than DM apart from rice milk which concurs with previous work [30,31]. It was found that PBMA that had a fat profile similar to “semi-skimmed” milk are lower in energy content to DM. Some PBMA include words like “semi” or “light” on the front-of-pack labeling, which could lead to a presumed energy content. For “semi-skimmed” and “between semi-skimmed and whole” fat profiles, there was a significant difference between dairy and PBMA in saturated fat and most PBMA (except for coconut milk and oat kefir) were lower in saturated fat. This is concurrent with the literature [30,48]. Nutritional guidelines in the United Kingdom advise consuming less saturated fat due to an increased risk of cardiovascular disease [44] so this is a potential benefit of PBMA.

Dairy milk contained more carbohydrate (g) per 100 mL than PBMA which was in line with previous research [30]; however, this difference was not significant. There was found to be a significant difference in median carbohydrate content between milks with soya, coconut, and almond milk having the lowest content. Carbohydrate content of lactose-free milk was significantly lower than cows milk. Lactose-free milk is often filtered to remove lactose present and contains the enzyme lactase which breaks down lactose and makes the milk easier to digest for people who are lactose intolerant.

Dairy milk contained significantly more total sugar (g) per 100 mL than PBMA. It was found that rice milk had a higher median total sugar content than all other milk. These findings concur with previous research [31]. Although significance could not be tested due to low sample sizes in this milk subtype, this finding may be important for dietitians helping patients living with diabetes manage their blood glucose levels. Almond and coconut milk were significantly lower in sugar than all other milk. It should be noted that PBMA may contain “added” or “free” sugars’ (instead of natural sugars found in DM), which the NHS advises reducing [45]. Clegg et al. [30] also found that PBMA contained more free sugars than DM. However, the median sugar in PBMA was 2.5 g/100 mL, so unless someone is drinking a lot of milk, the contribution toward the advised limit of 30 g “free sugar” per day will be low [45].

According to McCance and Widdowson dietary reference values [39], DM does not contain fiber, and this was reflected in the DMs collected, and concurs with previous research [30]. Ninety-five percent of PBMA contained fiber, which has various health benefits in the general population, for example, it supports gut health, and protects against chronic diseases such as cardiovascular disease, type 2 diabetes, and bowel cancer [46]. The National Diet and Nutrition Survey (NDNS) found that mean intakes of fiber were between 17.3 and 19.7 g in adults [47] which is below the recommended 30 g/d [46]. The mean amount of fiber in 100 mL PBMA is 0.5 g which equates to around 1.5 g per 250 mL glass or 4% of 30 g/d so PBMA could contribute to improving fiber intake, if only slightly.

Dairy milk contained significantly more protein than PBMA. The only comparable milk to cows’ milk in terms of protein content was soya milk which concurred with Medici et al. [31] findings. In the United Kingdom, most adults meet the reference nutrient intake for protein [49] and it is available from a variety of other dietary sources. However, for certain demographics, such as young children, those with lower incomes and poor diet diversity, a switch to PBMA may be inappropriate [50]. For example, a study using NDNS data found that adults aged over 75 y obtain 12.1% of their protein requirements from milk, yogurt, and cheese [30]. There is emerging evidence that DM has a range of indispensable amino acids, and that the quality of milk protein may be higher than of plant proteins [51].

In the analysis of subtypes with sufficient sample sizes, almond milk had a higher salt content than all other milks. This concurs with Medici et al. [31] who also found that both almond milk and combination milk have a higher salt content, an important finding for healthcare professionals to be aware of.

Dairy milk naturally contains a wide variety of micronutrients including calcium, phosphorus, potassium, iodine, vitamin B_2_, and vitamin B_12_ [26]. Of nonorganic PBMA, 87% were fortified with calcium, 1% phosphorus, 3% potassium, 34% iodine, 79% vitamin B_12_, and 56% vitamin B_2_. Clegg et al. [30] compared the nutritional content of PBMA (136) with DM in the United Kingdom and found that 100% PBMA were fortified with calcium, 7% with potassium, 7% with iodine, 57% with vitamin B_2_, and 88% with vitamin B_12_. Medici et al. [31] found that 76% of PBMA were fortified with calcium, 12% with iodine, 50% with vitamin B_2_, and 64% with vitamin B_12_. It is important to note that Medici et al. sample included milks from all over Europe. They discuss that there is a higher prevalence of organic varieties in Europe and that European regulations prohibit the fortification of organic foods and drinks [40]. Craig and Fresán [28] agree that fortification generally occurs more frequently for products sold in the United States than in Australia and Europe. However, in this study, even removing those PBMA labeled as “organic,” 8% PBMA were not fortified at all. Consumers and healthcare professionals need to be aware that products, especially those marketed as “organic,” may not have comparable micronutrients to DM, which could lead to deficiencies if these are not met by other dietary sources.

Although many of these nutrients can be sourced from other foods, it is important to consider that for nonmeat eaters the main dietary source of vitamin B_12_ and iodine in the United Kingdom diet is dairy [26,52]. From analysis of NDNS data, it was found that iodine intake was significantly lower in exclusive consumers of PBMA than cows’ milk consumers and they were classified as iodine deficient as opposed to cows’ milk consumers who had sufficient blood concentrations [52]. In addition to varying content, research has also found variation in the bioavailability of micronutrients in PBMA. Heaney et al. [53] found that the bioavailability of calcium in soya milk was lower than in DM. Walther et al. [54] discuss the antinutrient properties of plant-based milk, such as, phytic acid, which occur naturally in plants and can limit the bioavailability of micronutrients, such as calcium, magnesium, and iron. They suggest that further research into the digestibility and absorption of PBMA would be beneficial in comparing the two. Despite a low proportion of PBMA being fortified with some micronutrients, it was found that 83% of nonorganic PBMA were fortified with total vitamin D which concurs with other studies with a range of 71%–88% [30,31]. The United Kingdom Government recommends that the population should take 10 μg/d vitamin D supplementation during the autumn and winter [55] and fortified milk could help contribute toward this. The mean total vitamin D (in those PBMA that were fortified) was 0.848 μg/100 mL which equates to 21% of recommended daily intake in 1 250 mL serving of milk. Craig et al. [23] found that replacing 250 mL DM for fortified soya or oat milk per day vitamin D intake increased by 85%. There are currently no guidance/recommendations to manufacturers on the fortification of PBMA. Between 4% and 5% of PBMA are fortified with vitamin A; an important consideration for pregnant women, for whom the advice is to avoid taking supplements that contain vitamin A [56].

Although the nutritional profile of DM has remained consistent, there have been changes to PBMA in recent years. Wall et al. [37] looked at nutritional differences in PBMA between 2020 and 2023 and found that fat and saturated fat have increased 1.6% and 1.0%, respectively, and protein has increased by 3.5%; however, levels remain lower than DM. Despite being “free sugars,” sugar has decreased by 24.2% and fiber has increased by 43.9%. They found that fortification overall has improved from 57% to 78% with a higher proportion of PBMA fortified with iodine, calcium, and vitamin B_12_. However, between different products fortification is still highly variable with a proportion of PBMA unfortified.

### Product composition

The finding that PBMA vary in the amount of main ingredient, with almond and walnut milk on mean only containing 2% almonds or walnuts, respectively, is important to consider when choosing a milk based on health benefits of that key ingredient.

In this study, added ingredients in PBMA included salt (92%), sugar (26%), flavors (33%), stabilizers (66%), emulsifiers (24%), and acidity regulators (77%). Fructuoso et al. [57] collected data and created a “word cloud” of the most added ingredients to PBMA; highest were salt, sugar, tricalcium phosphate, and gellan gum. Drewnowski [58] found that 69% products had “added salt,” 53% had added sugar, and 90.1% contained flavors, gums, stabilizers, and preservatives. This study was conducted in the United States; however, this study also reported that 92% PBMA in the United Kingdom have added salt. It is possible that the smaller range of United Kingdom milks with added sugar reflects a preference for unsweetened milks in the United Kingdom. Of the PBMA not labeled as organic, 97% are classified as “ultraprocessed” under the NOVA classification which is in concordance with the SACN, compared with DM which is “unprocessed”. PBMA rely on added ingredients to enhance the sensory elements/improve shelf life as well as fortification with micronutrients. Studies have identified an association between higher consumption of ultraprocessed foods and adverse health outcomes [59]. It is unclear whether this association is due to the “processing” including the addition of ingredients that are chemically synthesized or because processed foods naturally tend to be higher in calories, saturated fat, salt, and/or sugar [35]. To date, there is limited evidence for a causal link between food processing and poor health outcomes and hence no government or dietary guidelines. Therefore, processed foods cannot be deemed as unhealthy or unsuitable for consumption and it is important to consider the diet as a whole [61,62]. It is anticipated that the joint working group of SACN and the Committee on Toxicity of Chemicals in Food, COT will offer some insights. Considering this, classifying products purely by the degree of processing or ingredients, and without considering their nutritional profile can lead to mixed messages to consumers. For example, in this study, milks with added soluble or insoluble fiber were classified as ultraprocessed under the NOVA criteria [33]. The concept that adding ingredients to enhance nutritional value can also negatively impact the NOVA score is complex and requires further research. Equally, PBMA may be consumed for medical reasons, for example, milk allergy and many people are choosing PBMA for environmental and moralistic reasons so guidance around the consumption of ultraprocessed foods should take these factors into account. Craig et al. [23] suggest that the NOVA criteria for ultraprocessed products should not include plant proteins, natural stabilizers, and vitamin and mineral mixes.

### Cost

It was found that PBMA are significantly more expensive than DM per 1 L which concurs with most previous research [30,63], however, conflicts with Glover et al. [32] who found that prices were similar across dairy and nondairy milks. A recent study has found that overall PBMA have reduced in price by 3% from July 20202 to March 2023, whereas DM has increased by 50%, narrowing the gap in price between dairy and PBMA [37]. Although PBMA tend to be sold in 1 L cartons, DM is accessible in various container sizes. If consumers were to purchase the largest available (∼3 L for some milk), there would likely be additional cost savings.

### Strengths and limitations

This study used a larger sample size than previous studies, collecting data for all milk from the top 10 United Kingdom supermarkets therefore limiting bias. The study collected data on kefir, a fermented milk drink that is made from kefir grains, as it is used in a similar way to milk and global sales increased at a compound annual growth rate of 3% from 2019 to 2023 and is projected 3.6% between 2024 and 2034 [38]. Previous studies focused on plain, unflavored varieties of PBMA and compared them to lower-fat dairy products [31]. Sweetened PBMA were included to allow comparison with the full range of DMs.

Data is current which is crucial in this expanding market of PBMA, and it offers a cost comparison per 1 L milk. Medici et al. [31] found a large range in nutrients between and within milk categories. This study stratified milk by fat profile so that more meaningful comparisons could be made. Nutritional information was obtained from supermarket websites/manufacturers or back-of-pack labeling. However, for DM, full micronutrient information was missing, and so food composition tables were used. New data has since been published on micronutrient values in DM [72]. Equally, both methods compare unfavorably with the reference standard of direct chemical analysis [68].

Milk subtype categories were excluded from statistical analysis where sample sizes were either <5 or where they were not available in all supermarkets (camel, rice, hazelnut, cashew, hemp, pea, walnut, Jersey, goats, kefir, oat kefir, coconut, and combination milks) despite being popular choices. The market share of PBMA should be further investigated to include these products.

The findings of this study focus on the statistically significant differences between 100 mL of DM and PBMA however do not consider additional dietary intake. We cannot therefore deduce the clinical importance of these findings on an individual level.

### Environmental impact

Environmental impact is an important consideration when discussing milk choice, given the growing consumer demand, on the basis that this may lower a person’s environmental footprint [13]. Obtaining the environmental impact of food and drink products is both complex and challenging. This study aimed to investigate the environmental impact of PBMA and DM in the United Kingdom using the Clark et al. [22] dataset. However, the analysis was considered not sufficiently robust to draw meaningful conclusions.

Some of the key issues include that not all environmental impacts are assessed for food and drink products; there can be trade-offs between different environmental objectives (that can be hidden in aggregated metrics); impacts are not contextualized; and data for some products are derived from secondary sources (not measured primary data).

The dataset is largely derived from product LCAs which although provides a consistent framework, does not adequately cover all environmental impacts of food products; does not account for site-specific impacts (at the farm level); and, being product oriented, tend not to account for different methods of production at the farm level. For example, within this dataset, LCA handles impacts such as GHG emissions well, but does not account for more local impacts, such as effects on wildlife populations and biodiversity. Some methods of dairy production (e.g. grass-fed systems) have significant positive impacts on ecosystem services and biodiversity [64,65]. Clark et al. [22] also highlight that environmental impacts do not account for post production, processing, packaging, and transportation which may affect the estimated scores for airfreighted produce for example. Additionally, the data are not normalized (contextualized). For example, what is the significance of differences in GHG emissions between products in relation to the scale of the environmental issue. To answer this, emissions need to be normalized, for example, expressing them relative to the emissions of an mean person (in the EU) or relative to planetary boundaries (i.e. a target value considered to be sustainable) [60,66,67].

The above briefly highlights some of the complexities involved in understanding the environmental impact of food and drink products. From the perspective of the consumer, this poses a significant challenge for purchasing decisions, which involves considering the cost of different products and their nutritional and environmental attributes. Integrating these criteria into purchasing decisions is difficult, so the addition of uncertainties in the data and potential trade-offs and synergies between the criteria presents a challenge that is yet to be resolved. Further research is needed to find solutions that will facilitate consumer purchasing decisions that are both healthier and more sustainable. Greater consideration/discussion of the limitations of secondary environmental impact datasets is warranted to ensure full transparency in this multidisciplinary research area. This research highlights the need for robust, primary research into the environmental impact of foods.

### Future research

We have explored a range of factors that influence the population in choosing a milk product. Future research could also investigate sensory factors such as texture and appearance [24], mouthfeel, and flavor [69] of milk. Additionally, research is needed to examine trends in cost and nutrient compositions over time as the trends of flexitarian diets continue to evolve. Future research could also examine the nutritional intakes of those who consume PBMA, in comparison with dietary reference values, to better contextualize overall nutritional contribution of milk in the diet. Further research is also required to explore the quality and bioavailability of nutrients, such as protein or calcium, in PBMA compared with DM. Emerging research on the benefits of fermented dairy products in reducing the risk of noncommunicable diseases such as diabetes, highlights the need for further investigation into fermented milks. While this study had a relatively small sample of these products, it was observed that there is growing availability of flavored kefir and fermented milks available.

The Food Standards and Information focus Group is currently working on draft guidance on how plant-based dairy products can be described in marketing and packaging and proposes a law against terms such as “plant-based alternative.”

### Implications for healthcare professionals

With an expanding market for plant-based diets and the variety of reasons that people are choosing to consume PBMA, healthcare professionals including dietitians and nutritionists will increasingly need to be prepared for discussions on nutritional and cost differences using evidence-based information. Dietitians are uniquely placed to deliver personalized dietary advice depending on a person’s nutritional status, medical history, and goals [70]. They will be able to tailor their advice about PBMA and guide people to ensure nutritional adequacy in their diet. For some, e.g. the frail elderly, advice may be opting for milk with a similar energy and protein content to dairy. For others, e.g. those looking to lose weight or manage type 2 diabetes advice may be choosing a milk lower in energy, saturated fat, and total sugar.

In conclusion, the nutritional compositions of PBMA and DM collected in this study in the United Kingdom are different. PBMA can be used in a similar way to DM; however, it is not an equitable nutritional replacement. PBMA contain fiber and vitamin D which DM does not and contain lower amounts of saturated fat (except coconut milk) than dairy. However, overall PBMA have a lower protein, carbohydrate, and energy content than dairy. With such a large variation in the fortification of PBMA, additional consideration may be warranted in consumers of PBMA to ensure recommended intake of micronutrients is met in the diet particularly vitamin B_12_, iodine, and calcium. This highlights a need for greater consistency in the fortification of PBMAs to ensure comparable composition. Greater transparency on organic product packaging would ensure consumer awareness regarding of the products nutritional profile. There does not appear to be 1 type of PBMA which matches most closely to DM. For example, soya milk is closest in protein, whereas oat is closest in carbohydrates. However, if a person is looking to increase their fiber, their vitamin D or decrease their saturated fat, they may wish to consider PBMA. There are also differences in cost with DM being less expensive than PBMA. Ninety-seven percent of PBMA (that are not labeled as organic) are classified as “ultraprocessed” under the NOVA classification. However, some added ingredients are perceived as being beneficial to health, for example, fiber. Further research on the potential health impacts of non-nutritive additives and the level of processing in PBMA may be warranted. The environmental impact of food is of growing concern to consumers. However, the analysis into environmental impact was considered not sufficiently robust to draw meaningful conclusions, highlighting the need for quality, primary research into the environmental impact of foods.

## Author contributions

The authors’ responsibilities were as follows – GKN: designed the research (development of overall research plan, and study oversight), conducted research (data collection), analyzed data, performed statistical analysis, and wrote the manuscript; KEE: designed the research (project conception) and supervised all aspects; RF: reviewed the manuscript and provided support throughout the study; and all authors: read and approved the final version of the manuscript.

## Funding

The authors reported no funding received for this study.

## Data availability

Available on request.

## Conflict of interest

The authors report no conflicts of interest.
