# Supplementary material for: Exploring the Nutritional Profile and Cost of Plant-Based Milk Alternatives Compared with Dairy Milk in the UK with Consideration of Environmental Impact Data
Source: Curr Dev Nutr. 2025 Apr 9;9(6):107436. doi: 10.1016/j.cdnut.2025.107436 (PMC12162003; doi:10.1016/j.cdnut.2025.107436)
Supplement: multimedia component 1 [file mmc1.docx]

**Exploring the nutritional profile and cost of plant-based milk alternatives versus dairy milk in the UK with consideration of environmental impact data.** Gemma K Nowson

**Supplemental material**

**Supplemental Table 1**: Median energy (kcal/100ml), total fat (g/100ml), saturated fat (g/100ml), plus the interquartile ranges for dairy and PBMA according to fat profiles. P values indicate significant differences in median nutrient between types of milk.

|  |  | **Nutrient** | | | | | | | | | |
| --- | --- | --- | --- | --- | --- | --- | --- | --- | --- | --- | --- |
|  |  | **Energy (kcal/100ml)** | | | **Total Fat (g/100ml)** | | | **Saturated fat (g/100ml)** | | | |
|  |  | **Type of milk** | | | | | | | | | |
|  |  | **Dairy (n=1-73)** | **PBMA (n=0-68)** | **p value** | **Dairy (n=1-73)** | **PBMA (n=0-68)** | **p value** | **Dairy (n=1-73)** | **PBMA (n=0-67** | **p value** |  |
|  |  | **Median (IQR)** | |  | **Median (IQR)** | |  | **Median (IQR)** |  |  |  |
| Fat profile (g/100ml) | 0.1-0.5 (skimmed) | 35 (4) | 38 (5) | / | 0.3 (0.4) | 0.5 (0.15) | / | 0.1 (0.03) | 0.4 (0) | / |  |
|  | 0.6-1.4 (between skimmed and semi Skimmed) | 41a | 23 (21.75) | / | 1a | 1.2 (0.2) | / | 0.6a | 0.2 (0.6) | / |  |
|  | 1.5-2 (semi skimmed) | 49 (4) | 42 (18) | <0.00 | 1.7 (0.2) | 1.6 (0.4) | 0.201 | 1.1 (0.1) | 0.2 (0.1) | 0.000 |  |
|  | 2.1-3.4 (between semi skimmed and whole) | 59 (7) | 53 (20) | 0.053 | 2.9 (0.1) | 2.5 (0.7) | 0.144 | 2 (0.4) | 0.3 (0.2) | 0.000 |  |
|  | 3.5-4 (whole) | 65.5 (2) | 49 (13) | <0.001 | 3.7 (0.1) | 3.5 (0.5) | 0.608 | 2.4 (0.1) | 0.5 (2.2) | 0.382 |  |
|  | 4.1-4.7 (between whole and Jersey) | 72.5 (/) | 60 (/) | / | 4.4 (/) | 4.3 (/) | / | 2.9 (0) | 0.6 (/) | / |  |
|  | 4.8-5.7 (Jersey) | 79.5 (7) |  | / | 4.95 (0.38) |  | / | 3.4 (0.33) | / | / |  |

**
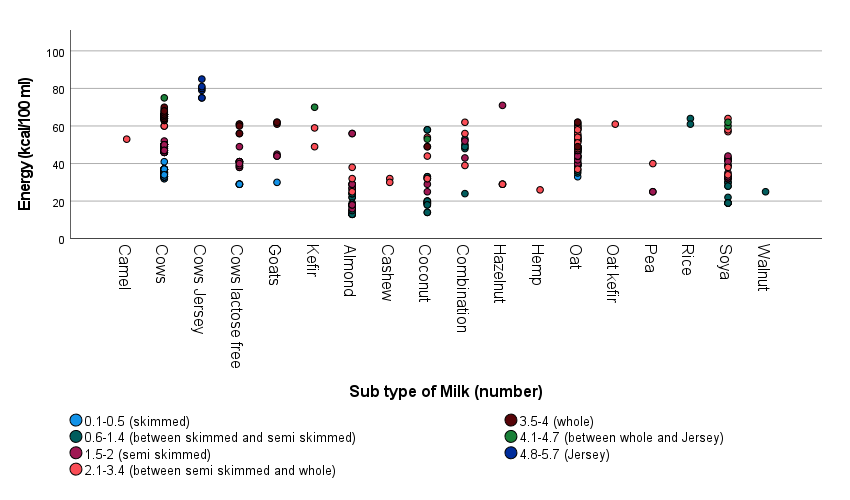
**

**Supplemental Figure 1:** Scatterplot of energy (kcal/100ml) for sub types of milk, according to fat profile

**
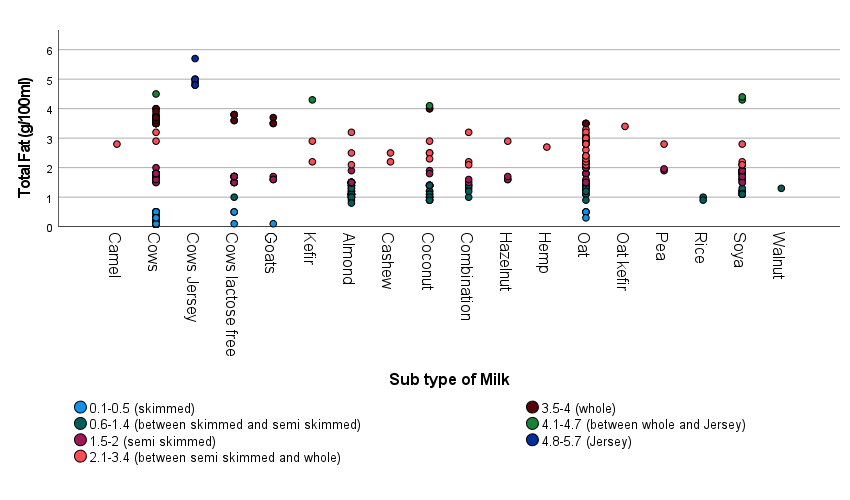
**

**Supplemental Figure 2:** Scatterplot of total fat (g/100ml) for sub types of milk, according to fat profile

**
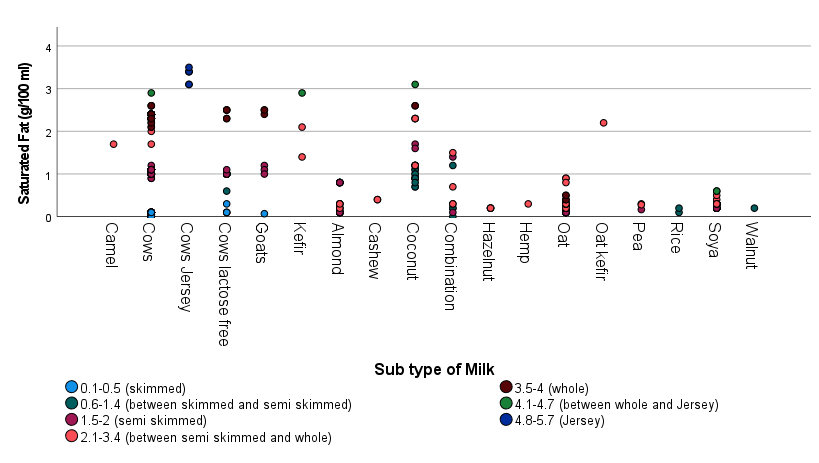
**

**Supplemental Figure 3:** Scatterplot of saturated fat (g/100ml ) for sub types of milk, according to fat profile

**Supplemental Table 2:** Median costs of all sub types of milk

| Milk sub type | N | Median cost per L (£) | IQR | Q1 | Q3 |
| --- | --- | --- | --- | --- | --- |
| **Dairy** | **332** | **1.19** | **0.59** | **0.91** | **1.50** |
| Camel | 1 | 13.83^a^ | 0 | 13.83 | 13.83 |
| Cows | 290 | 1.06 | 0.48 | 0.88 | 1.36 |
| Cows Jersey | 6 | 1.48 | 0.33 | 1.35 | 1.68 |
| Cows Lactose Free | 23 | 1.35 | 0.49 | 1.35 | 1.84 |
| Goats | 7 | 2.15 | 0.05 | 2.15 | 2.20 |
| Kefir | 5 | 5.20 | 1.9 | 4.70 | 6.60 |
| **PBMA** | **203** | **1.95** | **0.8** | **1.35** | **2.15** |
| Almond | 38 | 1.90 | 1 | 1.25 | 2.25 |
| Cashew | 2 | 2.67 | 0.33 | 2.50 | 2.83 |
| Coconut | 26 | 2.00 | 0.31 | 1.81 | 2.12 |
| Combination | 10 | 2.40 | 0.5 | 2.08 | 2.58 |
| Hazelnut | 4 | 2.30 | 0.41 | 2.09 | 2.50 |
| Hemp | 1 | 1.75^a^ | 0 | 1.75 | 1.75 |
| Oat | 73 | 1.98 | 0.48 | 1.62 | 2.10 |
| Oat Kefir | 1 | 7.40^a^ | 0 | 7.40 | 7.40 |
| Pea | 3 | 2.25 | 0.18 | 2.08 | 2.25 |
| Rice | 2 | 2.20 | 0 | 2.20 | 2.20 |
| Soya | 42 | 1.35 | 0.8 | 1.20 | 2.00 |
| Walnut | 1 | 2.15^a^ | 0 | 2.15 | 2.15 |
