# Supplementary material for: Exploring the Nutritional Profile and Cost of Plant-Based Milk Alternatives Compared with Dairy Milk in the UK with Consideration of Environmental Impact Data
Source: Curr Dev Nutr. 2025 Apr 9;9(6):107436. doi: 10.1016/j.cdnut.2025.107436 (PMC12162003; doi:10.1016/j.cdnut.2025.107436)
Supplement: multimedia component 2 [file mmc2.docx]

**Exploring the nutritional profile and cost of plant-based milk alternatives versus dairy milk in the UK with consideration of environmental impact data.** Gemma K Nowson

**Appendix A: An exploration into the environmental impact of plant-based milk alternatives compared with dairy milk in the UK**

**Results**

*Total Environmental Impact Score of plant-based milk alternatives compared with dairy milk*

**Figure A1:** Bar chart to show the median total environmental impact score’ (0 (lowest environmental impact) -100 (highest environmental impact)) per 100g milk. Error bars denote a semi-interquartile range (IQR/2). The red line shows the 50^th^ percentile impact (median) for dairy milk and the green line shows the 50^th^ percentile impact (median) for PBMA.

From Figure A1 it can be seen that dairy milk had a 75% higher median environmental impact score than PBMA and this was significant (p<0.0001). From the graph, almond milk has the highest median environmental impact and oat and soya milks have the lowest and pairwise comparisons showed that there were significant differences between oat and lactose free (p<0.05), oat and cows (p<0.0001), oat and goats (p<0.0001), oat and almond (p<0.0001), soya and lactose free (p<0.05), soya and cows (p<0.0001), soya and goats (p<0.005), soya and almond (p<0.0001), coconut and almond (p<0.005) and cows and almond (p<0.05).

Of note, if looking at the mean ‘total environmental impact score’, PBMA has a value of 1.97 per 100g milk, which is higher than dairy milk (1.55), likely skewed due to the high values for almond milk.

*Individual environmental indictors of plant-based milk alternatives compared with dairy milk*

The environmental impact of dairy milk, PBMA and all milk sub types for individual environmental indictors (greenhouse gas emissions, scarcity weighted water use, land use, aquatic eutrophication potential, acidification, and water use) per 100g of product) is shown in **Figure A2**

Dairy milk had a higher land use, GHGe, eutrophication, water scarcity, biodiversity and acidification than PBMA. Of note, some PBMA (almond, cashew, coconut, hazelnut, combination and walnut) had a negative median GHGe. Although dairy had a higher median water scarcity than PBMA, almond and cashew had the highest median water scarcity of all milks. Similarly, certain PBMA (almond and rice) had the highest median water use. However, we were unable to test whether these differences were significantly higher than dairy milk given that the data was not raw data.

**Figure A2:** Bar charts showing the 50^th^ percentile impact “median” of all sub types of milk (where available) for land use (L), GHG, eutrophication, water scarcity, acidification and water use per 100g sub type of milk. Error bars denote a semi-interquartile range (75^th^ percentile impact – 25^th^ percentile impact / 2). The red line shows the 50^th^ percentile impact for dairy milk and the green line shows the 50^th^ percentile impact for PBMA.
